# Effect of Heat Treatment on Microstructure and Tribocorrosion Performance of Laser Cladding Ni-65 WC Coating

**DOI:** 10.1155/2020/4843175

**Published:** 2020-08-20

**Authors:** Ze Liu, Eryong Liu, Shuangming Du, Congwei Li, Huiling Du, Yaping Bai

**Affiliations:** ^1^School of Materials Science and Engineering, Xi'an University of Science and Technology, Xi'an 710054, China; ^2^School of Materials Science and Chemical Engineering, Xi'an Technological University, Xi'an 710021, China

## Abstract

The Ni-65wt%WC cladding layers were prepared on the surface of Q235 using laser cladding technology, in which the effect of heat treatment on microstructure and tribocorrosion performance was investigated. The results showed that the coating is mainly consisted of Ni, WC, and W_2_C, and a significant diffusion phenomenon is formed between the interfaces of WC/Ni matrix, benefited for the improvement of bonding layer between WC/Ni-based matrixes. Meanwhile, the crystallization of WC particles after heat treatment was more obvious than untreatment; the Ni matrix grain size was also grown remarkable, leading to the lower hardness and weaker plastic deformation resistance of Ni-65wt%WC coating. And the erosion results showed that the wear rate of coating gradually decreased with heat treatment temperature increasing, while brittle WC was not suitable for high impact wear conditions. Furthermore, with the increase of heat treatment temperature, the reciprocating wear performance showed that the friction coefficient and wear rate of Ni-65wt%WC coating decreased. And the friction coefficient and wear rate of the coating (700°C) in 3.5% NaCl solution were 0.15 and 4.82 × 10^−8^ mm^3^·N^−1^·m^−1^, respectively. Therefore, the comprehensive comparison showed that Ni-65WC coating had better performance in low impact reciprocating testing under corrosion environment, and heat treatment was helpful to further improve the tribocorrosion performance of laser cladding Ni-65wt%WC coating.

## 1. Introduction

Q235 steel is the most commonly used metal material, which is widely used in the automobile industry, machinery manufacturing, and chemical industry [1–3]. However, low hardness and poor wear resistance limit its application in key friction movement parts. To solve these problems, researchers have prepared high hardness, antiwear, and corrosion-resistant coating on the surface of Q235 steel by laser cladding technology. And the composite coating has better development potential in the future due to saving a lot of precious metal materials and reducing production cost and energy consumption [4–7].

Laser cladding is a rapid melting and solidification process. Therefore, cracks caused by high thermal stress and microstructure stress in the preparation of the coating limit the application of the coating in engineering [8–11]. Heat treatment after cladding can not only effectively reduce the residual stress of the coating but also improve its mechanical properties [1, 12]. Lu et al. prepared a Ni60/H-BN coating on the surface of 304 stainless steel and heat-treated the coating at 600°C. The results show that the formation of carbides can effectively increase the hardness and wear resistance of the coating [13]. Li reported that the Ni-65WC coating heat-treated at 500°C could reduce the friction coefficient and improve the fracture toughness and wear resistance of the coating, obviously [14]. However, studying on the effect of postheat treatment to the microstructure, friction and wear properties of laser cladding composite coatings were less. Nickel-based alloys have been widely used in surface engineering because of their excellent corrosion resistance, good wettability, and relatively moderate price. Tungsten carbide has attracted much attention due to good chemical stability, thermal stability, and high hardness in the laser cladding wear-resistant composite coating in recent years.

In this paper, laser cladding technology is used to prepare Ni-65WC composite wear-resistant coating on the surface of Q235 steel, and the coating is postheat treated to reduce residual stress. The microstructure, friction and wear, and erosion-corrosion performance of the composite under the state of heat treatment are studied. This study provides a reference for improving the practical application of laser cladding to prepare composite wear-resistant coatings.

## 2. Experimental

### 2.1. Materials and Processing

In this study, the origin material was Q235 steel with dimensions of 100 mm × 60 mm × 20 mm. The raw Ni-based alloy powder had 10 wt% Cu, 0.95 wt% Si, 0.5 wt% B, 0.2 wt% Fe, and 88.35 wt% Ni. Then, the mixed powders (Ni-65 wt% WC) of Ni-based alloy powders (50-100 *μ*m) and WC powders (45-105 *μ*m) were used as the cladding material. The transition layer (Ni-Cr-B-Si) with a thickness of 1 mm was first evenly coated on the Q235 steel substrate. Then, the Ni-65WC powders were evenly spread through a preset powder process, and the thickness was approximately 1.5 mm. And they were next dried at 150°C for 6 h.

The multitrack-joined laser cladding processing was carried out by LSC-3000 laser heat treatment equipment. The optimum processing parameters are as follows: power—2500 W, spot diameter—6 mm, scanning speed—9 mm/s, and overlapping rate—50%. Next, the samples were cut into the size of 20 mm ×10 mm ×5 mm and treatment was at 500°C, 700°C, and 900°C (destress annealing, nitrogen protection) for 1 h, followed by furnace cooling.

### 2.2. Testing Detection Equipment and Methods

The microstructures of the cladding layers are observed by a scanning electron microscope (FE-SEM, JSM-6390A), and the crystal structure of the as-synthesized samples was detected by X-ray diffraction (XRD, Bruker) using Cu-K*α* radiation (*λ* = 1.54178 A) in the range of 20-90° with a scanning rate of 4°·min^−1^. The hardness of the cladding layers is tested by Vickers hardness tester (HV-1000) with a loading force about 1000 g for 15 s.

Furthermore, erosion performance of Ni-65wt%WC coating was tested by a self-assembled jet erosion testing machine. Quartz sand particles with a size of 400 mesh were mixed with water in a weight ratio of 1 : 4, and the erosion velocity, angle, and time were 5 m/s, 90°, and 30 min, respectively. The erosion-corrosion mass loss rate was calculated by the changes in weight.

The friction and wear performance of the cladding layers are detected by the friction and wear experiment (MFT-R4000) using Si_3_N_4_ ball (diameter 6 mm) as grinding materials. The testing parameters are as follows: wear time—120 min, load—50 N, the reciprocating frequency—20 mm/s, wear scar length—10 mm, and condition—3.5% NaCl solution. The wear rate was calculated by Equation (1), where *V* is the wear volume, measured by a 3D laser confocal microscope, *F* represents the load, and *S* represents the sliding distance. All tests were performed at least three times, and the average values of the friction coefficient and wear rate were recorded.

(1) $$\begin{array}{c} W_{loss}=\frac{V}{F\times S}. \end{array}$$

## 3. Results and Discussions

### 3.1. Microstructure and Mechanical Properties of Ni-65wt%WC Coating

**Figure 1:**
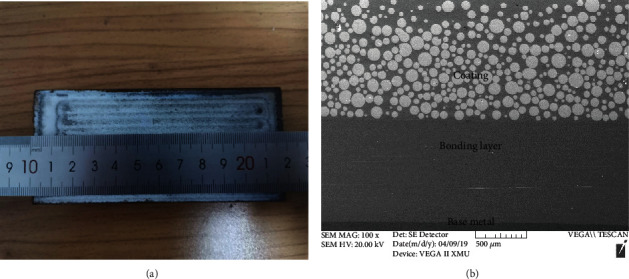
Macroscopic morphology (a) and cross-section microstructure (b) of Ni-65wt%WC coating.

The macroscopic morphology and cross-sectional morphology of Ni-65WC coating are shown in Figure 1.  Figure 1(a) shows that the cladding coating had no defects such as porosity and the white spherical WC particles which were relatively uniformly distributed in gray Ni base (Figure 1(b)). Furthermore, it also can be seen that the content of WC particles was higher under the cladding coating, which was attributed to the fact that the density of WC was much higher than that of Ni matrix. Meanwhile, the buckling or delamination was not observed on both the interface of the coating/bonding layer and the bonding layer/matrix. And the interface with a smooth sharp can be attributed to the low dilution or dissolution of alloying elements into the different layers.

**Figure 2:**
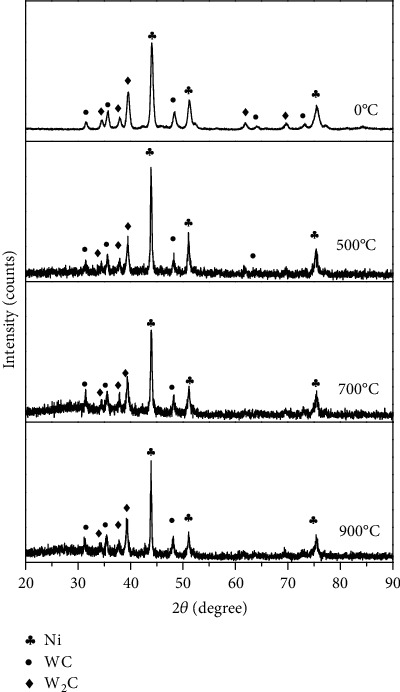
XRD pattern of Ni-65wt%WC coating after heat treatment at different temperatures.

XRD spectra of the Ni-65WC coating after heat treatment at different temperatures are shown in Figure 2. It can be seen that the sharp diffraction peaks in Ni-65wt%WC coating were found to be for Ni (04-0850), WC (51-0939), and W_2_C (20-1315). Due to the high-energy density laser and high cooling rate of laser cladding, the formation of intermetallic phases was beneficial to improve microhardness of the coating. Furthermore, XRD results of Ni-65wt%WC coating indicated that there were no new phases formed after heat treatment; only the diffraction peaks became narrower with the increase of temperature.

**Figure 3:**
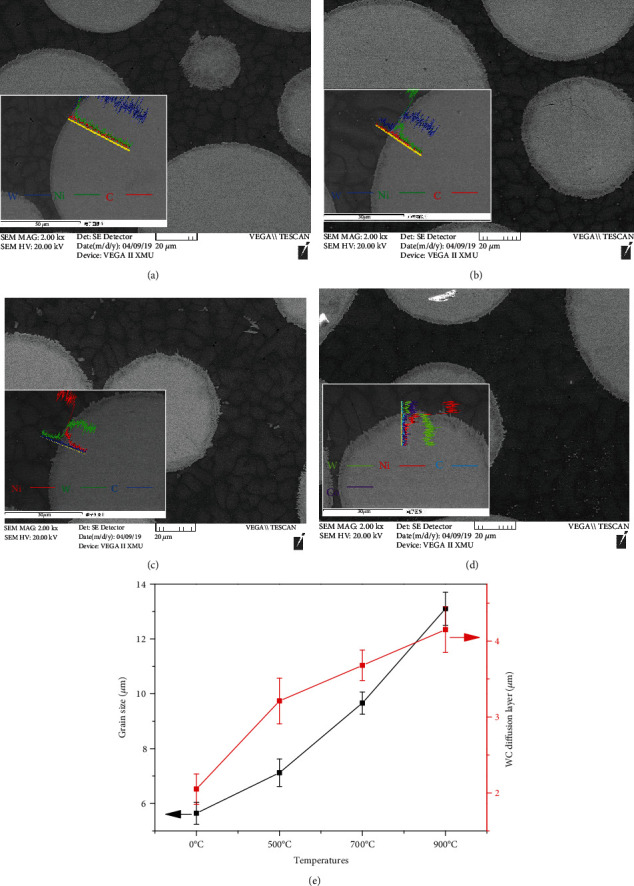
Cross-section microstructure and scanning of Ni/W interface of Ni-65wt%WC coating after heat treatment at different temperatures: (a) cladding coating, (b) 500°C, (c) 700°C, (d) 900°C, and (e) variation of grain size and the diffusion layer.

SEM images of Ni-65wt%WC coating after heat treatment at different temperatures are shown in Figure 3. The average grain size of Ni matrix and the width of the diffusion layer between WC particles and Ni alloy were calculated by image measure software, as shown in Figure 3(e). First, Figure 3(a) shows that the irregular globular grains of cladding Ni matrix were fine, dense, and uniform, and the average grain size of Ni-based coating was 5.64 *μ*m. After heat treatment, it can be seen that the average grain size of Ni matrix coating increased from 7.12 *μ*m (700°C) to 9.66 *μ*m (900°C). Meanwhile, the grain boundary of the spherical particles (Ni matrix) was fused during the heat treatment, which became almost oval particles. It was well known that the presence of Ni element will promote the decomposition of WC at high temperature. EDS results confirmed that the edge of WC particles dissolve, and the Ni content in the Ni/W interface diffusion layer continued to increase with the temperature increasing, indicating that the W and Ni elements at the Ni/W interface can diffuse with each other to form a bone-like diffusion layer during the heat treatment process.

**Figure 4:**
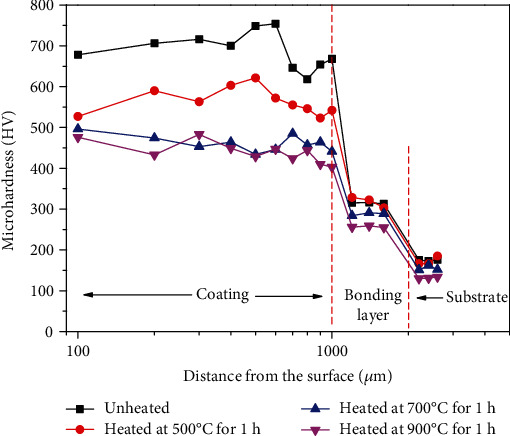
Microhardness of Ni-65wt%WC coating.

Figure 4 shows the microhardness of Ni-65WC coating after heat treatment. The hardness of the Q235 substrate was about 200 HV, and the average hardness of Ni-65wt%WC coating was 700 HV, which was attributed to the higher WC contents and the dissolved of WC particles in Ni-based alloy matrix. However, the average microhardness of Ni-65wt%WC coating after heat treatment significantly decreased from 600 HV at 500°C to 450 HV at 700°C. It can be seen that with the increased heat treatment temperature, the Ni-based grains of the coating grown (Figure 3(e)), and the resistance to dislocation motion decreased, resistance to deformation by external forces appeared to be reduced. Thus, the decrease of surface hardness was attributed to microstructure variation and the decomposition of WC particles.

### 3.2. Erosion Performance of Ni-65wt%WC Coating

**Figure 5:**
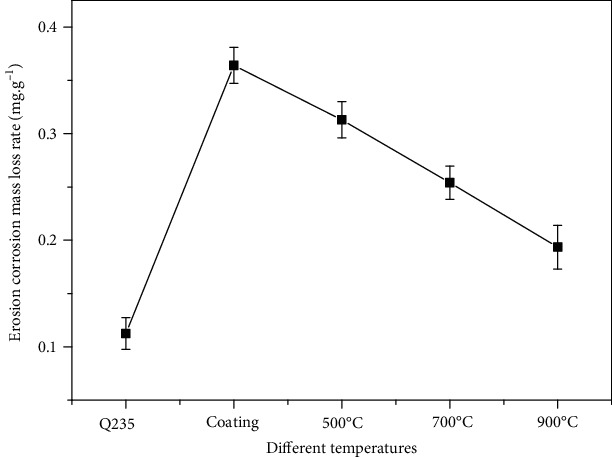
Erosion corrosion mass loss rate of Ni-65wt%WC coating.

Figure 5 shows the erosion performance of Ni-65wt%WC coating. With the increase of heat treatment temperature, the results showed that the erosion-corrosion mass loss rate of the untreatment coating decreased from 0.35 mg·g^−1^ to 0.15 mg·g^−1^ (900°C). However, the erosion-corrosion mass loss rate of Ni-65wt%WC coating was higher than that of the Q235 matrix, indicating that the coating had poor erosion resistance.

**Figure 6:**
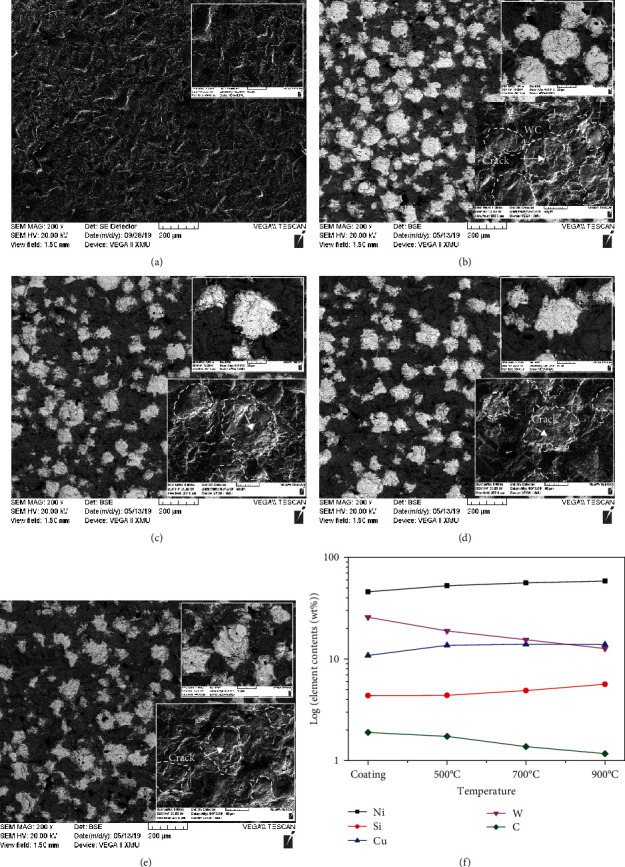
Erosion morphology of Ni-65wt%WC coating at different heat treatment temperatures: (a) Q235, (b) cladding coating, (c) 500°C, (d) 700°C, (e) 900°C, and (f) element distribution in erosion topography.

SEM images of the coating after erosion testing are shown in Figure 6. It could be seen that the surface of the Q235 matrix mainly consisted of chisel pit, confirming that the toughness Q235 was hard to resist the destructive effect of quartz sand particles. However, the damage of Ni-65wt%WC coating was more serious than that of the Q235 matrix, and the SEM image showed that almost all the spherical WC particles turn into irregular spherical particles. Meanwhile, many cracks, breaking, and peeling appeared on the surface of tungsten carbide, resulting in a large number of WC particles being broken [15–17]. Combined with the microstructure and erosion performance of Ni-65wt%WC coating, it could be concluded that the toughness phase was easier to resist the erosion of quartz sand particles by plastic deformation, while the brittle ceramic phase was smashed by quartz sand particles. After heat treatment, the width of the diffusion layer between WC/Ni-based alloys increased, which benefited from the combination of the coating. In this situation, the partially dissolved WC and Ni matrix formed a dense diffusion layer, which made it hard to be spall from the surface, and the residual WC particles played a role of erosion resistance with a metal matrix. Therefore, the erosion resistance of Ni-65wt%WC coating improved by heat treatment, especially at high temperatures with the width diffusion layer between WC/Ni matrixes. Furthermore, to further study the erosion mechanism of Ni-65wt%WC coating, EDS analysis was carried out on the erosion area of coating, as shown in Figure 6(f). The results showed that the erosion induced the decreases of W and C contents and increase of Ni, Si, and Cu on the erosion area of coating with the heat temperature, which can be owned by the strong combination between WC and Ni matrixes.

**Figure 7:**
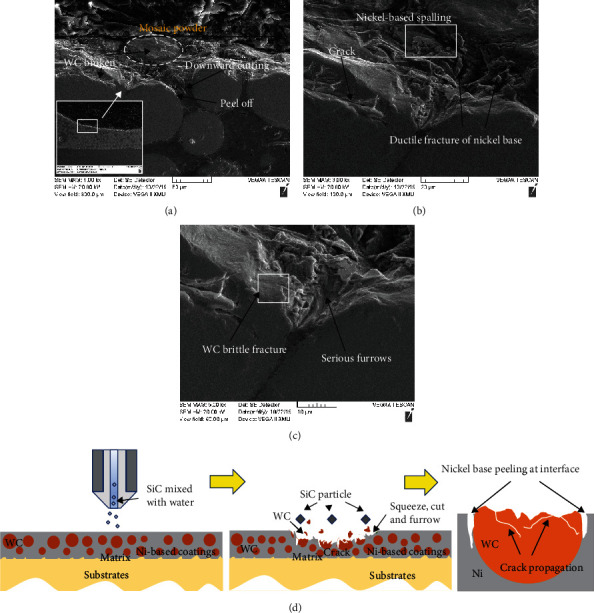
Section morphology (a–c) and schematic image of erosion mechanism (d) of Ni-65wt%WC coating under erosion wear condition.

The damage mechanism of Ni-65wt%WC composite coating under erosion is shown in Figure 7. Although WC particles and nickel matrix formed a good metallurgical combination in Ni-65wt%WC coating, the hard coating also showed poor erosion resistance under the high-speed erosion on 90°. During the erosion process, the cracks were easier to initiate and expand on brittle WC particles, and then, severe brittle fracture and spall appeared on WC particles, as shown in Figures 7(a) and 7(b). The Ni matrix damaged rapidly in the form of ductile fracture and then induced the lack of support of WC particles (Figure 7(c)).  Figure 7(d) indicates the schematic image of the damage mechanism of Ni-65wt%WC composite coating during the erosion process. It is well known that the interface of the WC/matrix was weaker than that of the Ni matrix; thus, crack initiation and propagation were easier to appear between the interfaces of WC particles and WC/matrix. Surface fatigue occurred on WC particles during erosion when a high-energy quartz sand particles were impacted on the surface, which was the first step under erosion. Subsequently, a crack initiated on the interfaces of WC particles or WC/matrix, and then, the crack gradually propagated with continuous erosion, resulting in serious spalling. Finally, it can be deduced that this kind of fatigue was likely to occur on Ni-65wt%WC coating with a weaker interface of WC/matrix and WC particles, such as cladding coating without heat treatment. Therefore, as the current results demonstrate, Ni-65wt%WC coating with brittle WC particles was hard to use in erosion resistance at 90° impact. In the future, it will be important to design application special functional materials integrated of strength and toughness in this condition.

### 3.3. Friction and Wear Performance of Ni-65 WC Coating under Reciprocating Tribocorrosion

**Figure 8:**
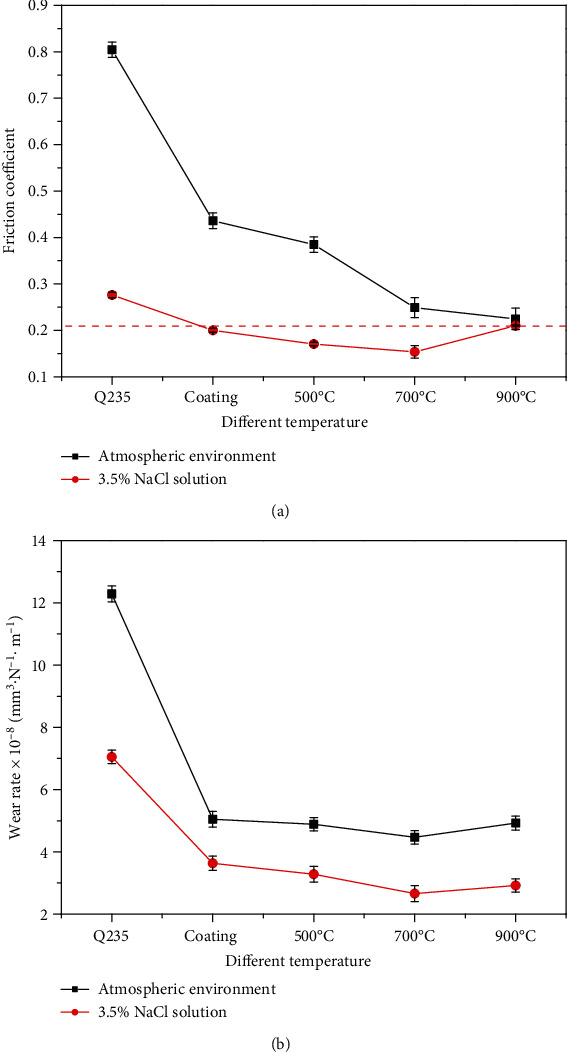
Friction coefficient (a) and wear rate (b) of Ni-65wt%WC coating under different environments.

After reciprocating tribocorrosion testing, Figure 8 shows the friction coefficient and wear rate of Ni-65wt%WC coating under the atmospheric environment and 3.5% NaCl environment, respectively. It can be seen that the friction coefficient of Ni-65wt%WC coating (0.436) was about 50% lower than Q235 steel substrate (0.804) under an atmospheric environment. Meanwhile, the wear rate of Ni-65WC coating (5.04 × 10^−8^ mm^3^·N^−1^·m^−1^) was about 60% lower than Q235 steel substrate (12.6 × 10^−8^ mm^3^·N^−1^·m^−1^). After heat treatment, the friction coefficient Ni-65wt%WC coating gradually decreased and the lowest wear rate was obtained for Ni-65wt%WC coating (4.82 × 10^−8^ mm^3^·N^−1^·m^−1^) at 700°C. Additionally, the friction coefficients and wear rates of Ni-65wt%WC coating at the 3.5% NaCl environment were significantly lower than the atmospheric environment. It can be seen that the Ni-65wt%WC coating after heat treatment at 700°C showed the lowest friction coefficient and wear rate; therefore, the results suggested that heat treatment reduces the friction coefficient and wear rate of Ni-65wt%WC coating under the atmospheric environment and 3.5% NaCl environment.

**Figure 9:**
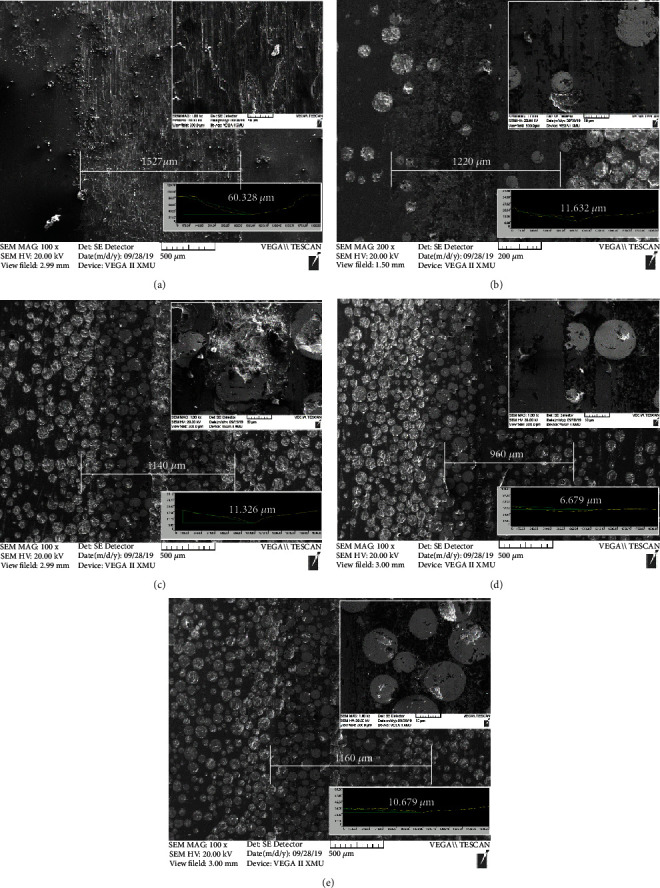
Wear scar morphology and depth of Ni-65wt%WC coating under air environments: (a) Q235, (b) cladding coating, (c) 500°C, (d) 700°C, and (e) 900°C.

Worn surface morphologies of Q235 steel and Ni-65wt%WC coating tested under the atmospheric environment are shown in Figure 9. The wear scar width (1220 *μ*m) of Ni-65wt%WC coating was significantly lower than that of the substrate (1527 *μ*m), and the wear scar width decreased with increasing temperature, and the minimum value (960 *μ*m) was obtained at 700°C. To study the wear mechanism of Q235 substrate and Ni-65wt%WC coating, the wear scar depth after wear testing under the atmospheric environment is also shown in Figure 9. According to the graph, the wear depth of the substrate (60.328 *μ*m) was about 5 times the coating (11.632 *μ*m), which proved that the Ni-65wt%WC coating had good wear resistance. Meanwhile, the wear depth of the coating was inversely proportional to the heat treatment temperature and the lowest was 6.679 *μ*m at 700°C. And it was found that the worn surface Q235 substrate was covered with tearing, microgroove, fracture, and spalling, proving the wear mechanism was dominated by abrasive and adhesive wear. Then, the worn surface of Ni-65wt%WC coating was covered with a microgroove, proving that the wear mechanism was dominated by abrasive wear. Additionally, it can be seen that the worn surface of Ni-65wt%WC coating consisted of gray Ni matrix and white WC particles, and the existence of WC was benefited for the wear resistance of Ni-based composition coating. Furthermore, the worn surface of Ni-65WC coating after heat treatment was different from cladding coating, it can be seen that the contents of WC particles on a worn surface were much higher than the cladding coating. Therefore, the wear resistance of the coating was improved by the increase of WC content, which was owing to the improvement of interface force between WC/Ni bases.

**Figure 10:**
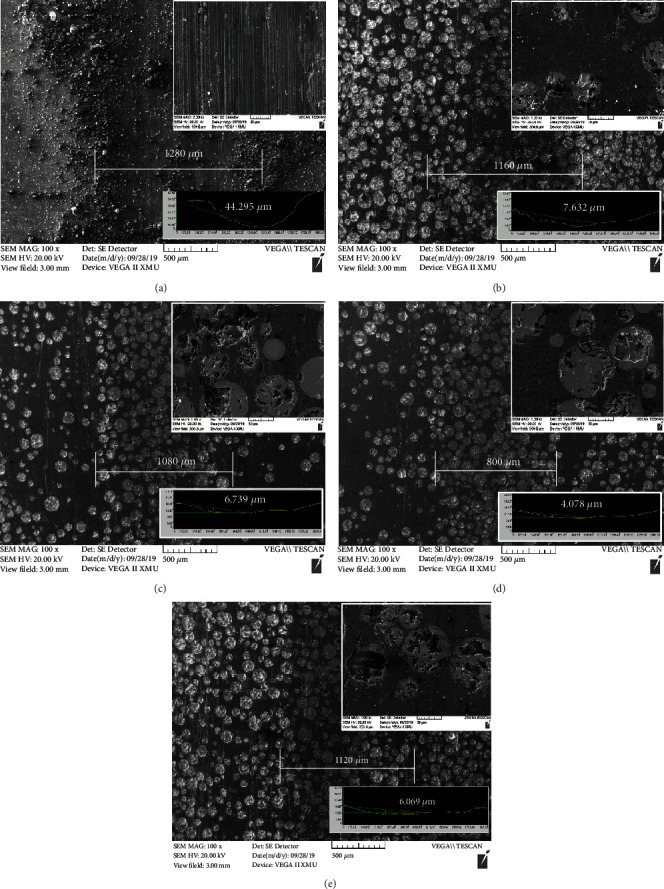
Wear scar morphology and depth of Ni-65wt%WC coating under 3.5% NaCl environments: (a) Q235, (b) cladding coating, (c) 500°C, (d) 700°C, and (e) 900°C.

Worn surface morphologies of Q235 steel and Ni-65wt%WC coating tested under a 3.5% NaCl environment are shown in Figure 10. The relationship between the width and depth of the wear scars with the increased heat treatment temperature was the same as the atmospheric environment. Meanwhile, the worn surface Q235 substrate was covered with microgroove and wear debris, indicating that the wear mechanism was abrasive wear. And the worn surface of the coating was smooth and accompanied by microgroove, confirming that the main wear mechanism was also dominated by abrasive wear. The width of the worn surface became narrower with heat treatment temperature, while being the lowest (800 *μ*m) at 700°C. Therefore, it can be concluded that heat treatment was benefited for improving the wear resistance of the Ni-65wt%WC coating.

**Figure 11:**
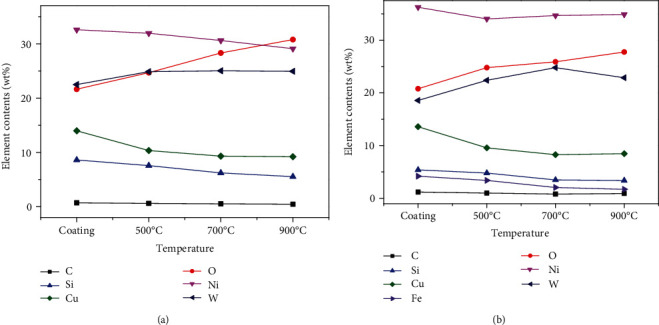
Chemical composition of wear scar of Ni-65wt%WC coating under different conditions: (a) air environments and (b) 3.5% NaCl environments.

The chemical composition of wear scar of Ni-65wt%WC coating under the atmospheric environment and 3.5% NaCl environment was tested by EDS, as shown in Figure 11. Under the atmospheric environment, the results indicated that the contents of Ni, Cu, and Si decreased and the contents of the W and O element increased on the worn surface of Ni-65wt%WC coating before and after heat treatment. Thus, it can be deduced that the contents of WC and oxide were higher on the worn surface of Ni-65wt%WC coating after heat treatment, which was beneficial for antifriction and wears resistance. Under the 3.5% NaCl environment, the contents of the W element on the worn surface of Ni-65wt%WC coating after heat treatment were significantly higher than those of the cladding state. Meanwhile, the contents of the W element on Ni-65wt%WC coating after heat treatment increased first and then decreased at the higher temperature, confirming that the contents of Ni and O elements increased continuously with temperature. According to the results of Wang Xiang [18], it can be deduced that Ni and O meant the existence of Nickel hydroxide. Hence, the increase of Nickel hydroxide and the decrease of WC deteriorated the tribological properties of Ni-65wt%WC coating.

**Figure 12:**
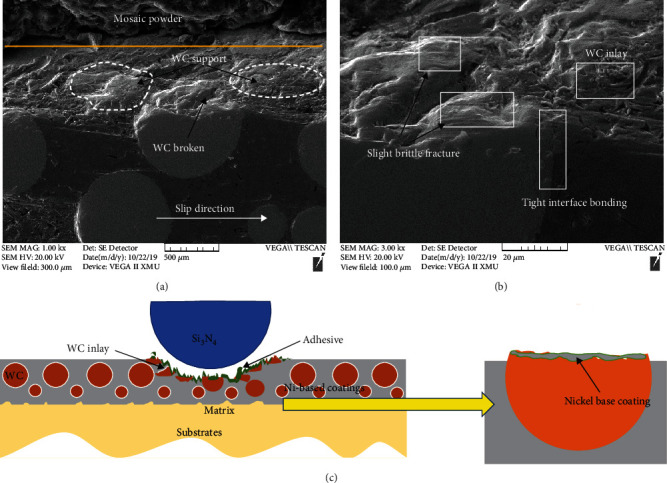
Section morphology (a, b) and schematic image of wear mechanism (c) of Ni-65wt%WC coating under reciprocating tribocorrosion condition.

To study the wear mechanism of Ni-65wt%WC coating under reciprocating tribocorrosion condition, the cross-section morphology and schematic diagram of worn surface of Ni-65wt%WC coating after heat treatment at 700°C are shown in Figures 12(a)–12(c). During the reciprocating wear process, the coating surface was continuously removed by applying a repeated load, resulting in partial spalling of tungsten carbide. Secondly, the soft nickel base was adhered to the surface of tungsten carbide under the repeated driving of grinding ball, preventing the spalling of WC. Therefore, WC particles remained in the subsurface had a significant antiwear effect. Compared with erosion Ni-65wt%WC coating, the damage of WC was much higher during erosion wear, indicating that brittle WC particles were suitable for antiwear under low impact conditions. Therefore, it can be deduced that WC particles only play effectively supporting and strengthening the role of Ni-based compositions coating under reciprocating wear conditions, and the performance of Ni-based composition coating under severe erosion condition was deteriorated.

## 4. Conclusions

1. The Ni-65wt%WC coating is prepared on Q235 steel by laser cladding technology, which mainly consisted of Ni, WC, and W_2_C. After heat treatment, the interaction dendrite structure is formed between the interfaces of WC/Ni matrix. Meanwhile, the grain size of Ni matrix grown leads to the lower hardness and weaker plastic deformation resistance of Ni-65wt%WC coating
2. With the increase of heat treatment temperature, the erosion rate of Ni-65wt%WC coating tested with quartz sand particle is lower at a higher temperature, which is enhanced by the stronger interface adhesion between WC/Ni matrixes
3. The Ni-65wt%WC coating shows excellent performance on reciprocating tribocorrosion under 3.5% sodium chloride condition, owning to the higher antiwear effect of hard WC under low impact condition

## Data Availability

The [DATA TYPE] data used to support the findings of this study are included in the article.
